# Accuracy, potential harm, weight stigma, and commercial influence in short-form social media content on incretin-based therapies for obesity

**DOI:** 10.3389/fendo.2026.1844311

**Published:** 2026-10-02

**Authors:** Yeo-Jun Yoon, Amanda Velazquez, Sehej-Leen Parmar, Katayoun Lotfi, Denise Wong, Cagney Cristancho, Katherine Salim, Seongwon Jeon, Sangmo Hong, Hyungoo Shin, Caroline M. Apovian, Dong Wook Kim

**Affiliations:** 1 Harvard T.H. Chan School of Public Health, Boston, MA, United States; 2 Jim and Eleanor Randall Department of Surgery, Cedars Sinai Medical Center, Los Angeles, CA, United States; 3 Harvard Medical School, Boston, MA, United States; 4 Palo Alto Medical Foundation, Palo Alto, CA, United States; 5 Section of Endocrinology, Diabetes, Nutrition, and Weight Management, Department of Medicine, Boston Medical Center, Boston, MA, United States; 6 Center for Weight Management and Wellness, Division of Endocrinology, Diabetes & Hypertension, Brigham and Women’s Hospital, Boston, MA, United States; 7 Hanyang University College of Medicine, Seoul, Republic of Korea

**Keywords:** digital health, GLP-1 receptor agonists, incretin-based therapy, obesity, social media, TikTok, YouTube

## Abstract

**Background:**

Short-form video platforms such as TikTok and YouTube Shorts are major sources of health information, yet little is known about the reliability of what they carry on incretin-based therapies for obesity. We assessed the accuracy, safety, obesity-related stigma, and commercial nature of short-form videos on these medications.

**Methods:**

We conducted a cross-sectional content analysis on June 15, 2025. Videos were retrieved for six medication keywords on TikTok and YouTube Shorts, and the 100 most-viewed videos within each platform’s retrieved pool were included (n = 200). Four physicians certified by the American Board of Obesity Medicine each coded a non-overlapping subset for clinical accuracy, hazard potential, obesity stigma, and commercial influence. A fifth senior physician reviewed all videos and issued final ratings. Six TikTok videos became unavailable before review, leaving 194 for content analysis.

**Results:**

TikTok videos had higher median view counts (964 650 [Q1–Q3 615 350–1 525 000] vs 24 526 [7 036–104 569], *p* < 0.001) and higher raw like and comment counts. Normalizing to views changed the picture. Likes per 1000 views did not differ (24.7 vs 20.4, p = 0.069), and comments per 1000 views were higher on YouTube Shorts (1.2 vs 0.8, p < 0.001), where videos were also older (720 vs 221 days, p < 0.001). Clinical accuracy did not differ significantly (23.4% [95% CI 16.0–32.9%] vs 39.0% [95% CI 30.0–48.8%], p = 0.062). Hazard potential was more common on TikTok (31.9% vs 17.0%, p = 0.015), but this came from promotion of compounded products, four times more frequent on TikTok (14.9% vs 4.0%, p = 0.009). Restricted to directly unsafe clinical content, the platforms did not differ (20.2% vs 13.0%, p = 0.176). Obesity stigma (p = 0.118) and commercial influence (p = 0.438) were comparable.

**Conclusion:**

Most short-form videos about incretin-based obesity therapies carried no accurate clinical information, and accurate videos drew only 24% of cumulative views. Promotion of compounded products clustered on TikTok and accounted for the platform difference in hazard potential. Evidence-based, bias-free communication is needed if these platforms are to educate the public about obesity pharmacotherapy.

## 1 Introduction

Glucagon-like peptide-1 receptor agonists (GLP-1 RAs) have become a primary option in obesity pharmacotherapy (1). Liraglutide (Saxenda, Novo Nordisk, Bagsvaerd, Denmark) received FDA approval in 2014 as the first GLP-1 RA for chronic weight management, followed in 2021 by semaglutide (Wegovy, Novo Nordisk) (2). In November 2023 the FDA approved tirzepatide (Zepbound, Eli Lilly, Indianapolis, IN, USA), a dual agonist of GLP-1 and glucose-dependent insulinotropic polypeptide (GIP) (3, 4). Public awareness and demand have grown steeply since, and prescription volumes and national spending have risen with them (5–7).

Because tirzepatide acts at both the GIP and GLP-1 receptors, it is not a selective GLP-1 RA. We use “incretin-based therapies” for the class as a whole and reserve “GLP-1 RA” for the selective agents.

These platforms distribute the same videos into very different regulatory environments. The United States is one of only two countries that permit direct-to-consumer prescription drug advertising. The United Kingdom and Australia prohibit it outright and regulate promotional health claims through the MHRA and the TGA. A video that is lawful promotion in one country may breach advertising law in another, and platform moderation does not track those boundaries. This bears on how our findings should be read, since most videos in our sample were made in the United States (**Supplementary Table 3**).

Social media has become a major source of health information, particularly for younger audiences who turn to TikTok and YouTube Shorts for guidance on diet and weight management (8, 9). The top 100 TikTok videos under the hashtag #Ozempic have together drawn more than 70 million views (10). Drug shortages that followed viral posts about semaglutide’s weight-loss effects show how quickly this content spills into clinical reality (11). The quality of what circulates varies widely, since much of it comes from non-experts rather than clinicians (12–14). Exposure to weight-related and diet-focused messaging on social media can reinforce weight-normative ideals and unhealthy behavior (15). Content from creators without medical credentials also tends to reach more people than expert-led communication does (16, 17).

Scholarly work has not kept pace with any of this. Only two published studies have analyzed semaglutide-related content on TikTok, both limited to videos tagged #Ozempic or #Semaglutide (10, 18). We analyzed short-form video content on incretin-based obesity therapies across TikTok and YouTube Shorts, assessing the accuracy, safety, and commercial character of what reaches viewers.

## 2 Materials and methods

### 2.1 Data collection

We set out to capture short-form video content on incretin-based obesity therapies. Six keywords were selected, covering the brand and generic names of the agents approved or widely used for weight management at the time of collection. These were Semaglutide, Wegovy, Ozempic, Tirzepatide, Zepbound, and Mounjaro. Broader tags such as #GLP1 or #GLP1weightloss were not used, because they return large volumes of content tied to no particular medication, including supplements marketed as natural GLP-1 products, which would leave the denominator of medication-specific content undefined. Liraglutide was not searched separately. Its brand names were rarely used in short-form content by 2025, and it has been largely displaced in practice by semaglutide and tirzepatide.

Data were collected on June 15, 2025 from the desktop websites of both platforms. Each keyword was queried on TikTok as a hashtag, for example “#Semaglutide,” and on YouTube Shorts as the keyword combined with “#shorts.” A new TikTok account was created for the study, private browsing was enabled, and location services were disabled, all to limit personalization of the results.

Search results were screened in view order until roughly 50 unique videos per keyword had been retrieved. The same video is often returned by more than one medication keyword, so records were consolidated by video identifier. Each video was assigned to the keyword under which it first appeared, and any additional matching keywords were recorded. This left a unique pool of 289 TikTok videos and 263 YouTube Shorts videos (n = 552). Videos were then ranked by view count, and the 100 most-viewed within each platform pool entered the final dataset (n = 200) (**Figure 1**). The pool was built from six medication keywords rather than from the platforms at large. Our sample therefore represents the most-viewed content those searches surfaced and should not be read as representative of all obesity-related content on either platform. **Supplementary Table 1** gives the number of included videos per keyword. Only publicly accessible, English-language videos were analyzed. A video was eligible if it discussed the medication in any context. We did not exclude content focused on type 2 diabetes rather than weight management, since the two indications overlap in public discourse and a viewer searching a brand name meets both. Extracted metadata covered view count, likes, comments, shares, upload date, and duration in seconds.

**Figure 1:**
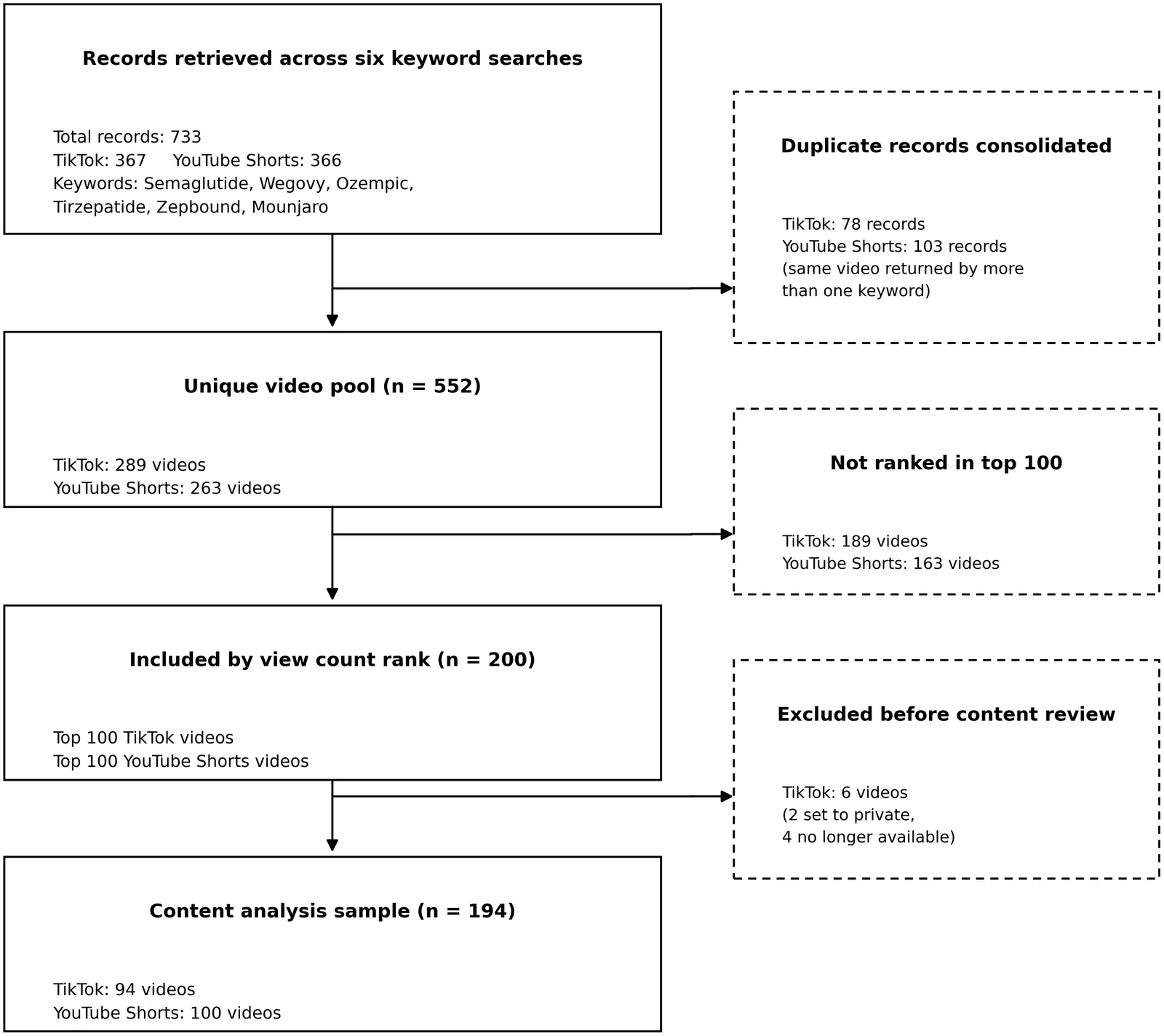
Flowchart of the video selection process for short-form videos on incretin-based obesity therapies.

### 2.2 Video review and evaluation

Five obesity medicine physicians took part in the evaluation, all fellowship-trained and certified by the American Board of Obesity Medicine. They worked from a written coding guide that defined each domain and its response options, piloted on a small set of videos before coding began. Four reviewers each coded a non-overlapping subset of 25 videos per platform, so every video received one initial rating. The fifth senior reviewer then viewed all 194 videos, compared each initial rating against the guide, and issued the final rating. Where his assessment differed from the initial rating, his rating stood. No video was coded twice, and formal inter-rater agreement could not be calculated.

Four domains were assessed. Clinical accuracy asked whether the medical information presented was evidence-based. Hazard potential covered information that, if acted on, could plausibly prompt a behavioral change leading to direct medical harm. Obesity stigma covered weight-related bias, negative body image, and shaming expressions. Commercial influence covered explicit or implicit promotion of a product, clinic, influencer brand, or service, including sponsorships, discount codes, and external links.

Clinical accuracy was rated Accurate, Inaccurate, or Insufficient. Insufficient marked a lack of adequate clinical information rather than the presence of incorrect information. Inaccurate content was assigned to one of seven mutually exclusive subtypes. These were distorted outcomes based on anecdotal experience, dosing or administration errors, false or exaggerated adverse effects, misrepresentation of indication or efficacy, misrepresentation of mechanism or data, non-evidence-based self-management recommendations, and promotion of compounded medications. Where a video carried more than one kind of inaccuracy, the reviewer recorded the dominant one.

We defined compounded medications as GLP-1 RA or GIP/GLP-1 RA preparations obtained from compounding pharmacies or other non-manufacturer sources rather than dispensed as an FDA-approved branded product. This included peptide preparations sold for research use only and products marketed as natural or herbal GLP-1 alternatives. Videos promoting such products were classified as hazard-positive *a priori*, on the grounds that these preparations undergo no FDA review for potency, purity, or sterility. That rule was applied by definition rather than case by case. We therefore report hazard potential under the composite definition and again restricted to directly unsafe clinical content, and report the composition of hazard-positive videos in the Results.

### 2.3 Statistical analysis

Analyses were run in Python version 3.10.16 (Python Software Foundation, Wilmington, DE, USA) using the pandas, numpy, statsmodels, and scipy libraries. Video characteristics and coding outcomes were summarized descriptively. Continuous variables were non-normally distributed on Shapiro-Wilk testing and are reported as medians with first and third quartiles. Categorical variables are reported as frequencies and percentages. Platforms were compared with the Mann-Whitney U test for continuous variables and the chi-squared test without continuity correction for categorical variables. Associations are expressed as odds ratios (ORs) with 95% confidence intervals (CIs). Clinical accuracy runs from Accurate through Insufficient to Inaccurate, so we also fitted a proportional-odds model and report cumulative ORs at each cut point. Engagement was analyzed as raw counts and again as likes and comments per 1000 views, since raw counts scale with audience size. A two-sided p value below 0.05 was treated as significant. We made no adjustment for multiple comparisons, and the four domain-level tests should be read with that in mind.

## 3 Results

### 3.1 Descriptive statistics

The metadata analysis covered 200 videos, 100 from each platform, drawn from 92 distinct creators on TikTok and 73 on YouTube. TikTok videos carried far higher raw engagement, with greater views, likes, and comments (all *p* < 0.001, **Table 1**). Those raw differences track audience size rather than audience response. Normalized to views, likes per 1000 views did not differ between platforms (24.7 vs 20.4, p = 0.069), and comments per 1000 views were higher on YouTube Shorts (1.2 vs 0.8, p < 0.001). The YouTube videos were also considerably older, at a median of 720 days against 221 on TikTok (p < 0.001), so TikTok videos had gathered far more views in far less time. Median duration did not differ (p = 0.34), but the two distributions are not comparable. Every YouTube Shorts video ran 60 s or less. Thirty-nine TikTok videos exceeded that, and the longest ran 432 s.

**Table 1:** Comparison of video metrics between TikTok and YouTube shorts.

Video metric	TikTok (n = 100)	YouTube Shorts (n = 100)	p value
Creators	92	73	N/A
View count	964 650 (615 350–1 525 000)	24 526 (7 036–104 569)	<0.001
Like count	24 600 (12 775–47 725)	427 (217–2 108)	<0.001
Comment count	888 (509–1 665)	40.5 (15–139)	<0.001
Likes per 1000 views	24.7 (16.6–42.4)	20.4 (14.4–31.1)	0.069
Comments per 1000 views	0.8 (0.5–1.3)	1.2 (0.8–2.5)	<0.001
Duration (s)	35.0 (16.0–104.8)	45.5 (23.5–58.0)	0.34
Video age at collection (days)	221 (117–391)	720 (454–884)	<0.001

Values are median (Q1–Q3). Missing like counts (n = 1) and comment counts (n = 2) on YouTube Shorts were treated as zero. All YouTube Shorts videos ran 60 s or less, while 39 TikTok videos exceeded 60 s, the longest at 432 s.

### 3.2 Content analyses

Six TikTok videos could not be reached at the time of content review, two set to private and four no longer available. Metadata analyses therefore rest on all 200 videos and content analyses on 194, comprising 94 TikTok and 100 YouTube Shorts videos. All six missing videos came from TikTok, which slightly reduces the TikTok denominator.

**Table 2** summarizes the four domains. Of the 94 TikTok videos analyzed, 23.4% (95% CI 16.0-32.9%) were rated clinically accurate against 39.0% (95% CI 30.0-48.8%) of YouTube Shorts (**Figure 2A**). The difference across the three accuracy categories was not statistically significant (p = 0.062), and the proportional-odds model gave a cumulative OR of 1.64 (95% CI 0.97-2.77) for less accurate content on TikTok (p = 0.066). The two cut points behaved differently. TikTok videos were less likely to be rated Accurate rather than Insufficient or Inaccurate (OR 2.09, 95% CI 1.12-3.91, p = 0.019), while the contrast between Inaccurate and everything else was not significant (OR 1.38, 95% CI 0.78-2.44, p = 0.272). The gap sits at the threshold of providing any accurate clinical information, not along a gradient of worsening inaccuracy.

**Table 2:** Distribution of clinical accuracy, hazard potential, obesity stigma, and commercial influence.

Domain	TikTok (n = 94)	YouTube Shorts (n = 100)	OR [95% CI]	p value
Clinical accuracy				0.062
Accurate	22 (23.4)	39 (39.0)	–	
Insufficient	28 (29.8)	22 (22.0)	–	
Inaccurate	44 (46.8)	39 (39.0)	–	
Proportional-odds model	–	–	1.64 [0.97–2.77]	0.066
Cut 1: Accurate vs Insufficient or Inaccurate	–	–	2.09 [1.12–3.91]	0.019
Cut 2: Inaccurate vs Accurate or Insufficient	–	–	1.38 [0.78–2.44]	0.272
Hazard potential (composite)	30 (31.9)	17 (17.0)	2.29 [1.16–4.51]	0.015
Directly unsafe clinical content	19 (20.2)	13 (13.0)	1.70 [0.78–3.66]	0.176
Promotion of compounded products	14 (14.9)	4 (4.0)	4.20 [1.33–13.27]	0.009
Obesity stigma	13 (13.8)	7 (7.0)	2.13 [0.81–5.60]	0.118
Commercial influence	17 (18.1)	14 (14.0)	1.36 [0.63–2.93]	0.438

Values are number (%). Odds ratios are for the presence of each domain on TikTok versus YouTube Shorts. The composite hazard definition counts a video as hazard-positive if it carried directly unsafe clinical content or promoted a compounded product. The two component rows are not mutually exclusive and are detailed in **Table 3**. Confidence intervals for proportions have been removed from this table and are reported in the text where relevant.

Hazard potential was more frequent on TikTok, at 31.9% (95% CI 23.4-41.9%) against 17.0% (95% CI 10.9-25.5%) on YouTube Shorts (OR 2.29, 95% CI 1.16-4.51, p = 0.015). **Figure 2B** and **Table 3** breaks these videos down. Of the 30 hazard-positive TikTok videos, 16 carried directly unsafe clinical content, 11 qualified only through promotion of a compounded product, and 3 carried both. On YouTube Shorts, 13 of 17 carried directly unsafe content and 4 qualified only through compounded promotion. Restricting hazard to directly unsafe clinical content removed the platform difference (20.2% vs 13.0%, OR 1.70, 95% CI 0.78-3.66, p = 0.176). Promotion of compounded products was about four times more common on TikTok (14.9% vs 4.0%, OR 4.20, 95% CI 1.33-13.27, p = 0.009). The excess hazard on TikTok therefore comes from compounded product promotion rather than from a broader excess of unsafe advice. Obesity stigma (p = 0.118) and commercial influence (p = 0.438) did not differ between platforms.

**Table 3:** Composition of hazard-positive videos.

Hazard classification	TikTok (n = 30)	YouTube Shorts (n = 17)
Directly unsafe clinical content only	16 (53.3)	13 (76.5)
Promotion of compounded products only	11 (36.7)	4 (23.5)
Both	3 (10.0)	0 (0.0)

Values are number (%) of hazard-positive videos on each platform. Promotion of a compounded product was classified as hazard-positive a priori.

**Table 4** gives the thematic subtypes of inaccurate content. Promotion of compounded GLP-1 RAs (29.5%) and anecdotal distortions (18.2%) led on TikTok, while false or exaggerated adverse effects predominated on YouTube Shorts (23.1%). Four YouTube Shorts videos rated Inaccurate received no subtype and appear as unclassified. Obesity stigma was present in 13 TikTok videos and 7 YouTube Shorts, and commercial influence in 17 and 14 videos respectively.

**Table 4:** Thematic subtypes of inaccurate TikTok and YouTube shorts videos.

Thematic subtype	TikTok (n = 44)	YouTube shorts (n = 39)
Promotion of compounded GLP-1 RA	13 (29.5)	3 (7.7)
Distorted outcomes based on anecdotal experience	8 (18.2)	7 (17.9)
Dosing or administration error	6 (13.6)	5 (12.8)
False or exaggerated adverse effects	6 (13.6)	9 (23.1)
Misrepresentation of indication or efficacy	4 (9.1)	4 (10.3)
Misrepresentation of mechanism or data	4 (9.1)	5 (12.8)
Non-evidence-based self-management recommendations	3 (6.8)	2 (5.1)
Not classified	0 (0.0)	4 (10.3)

Values are number (%) of videos rated Inaccurate on each platform. Subtypes are mutually exclusive. Where a video carried more than one kind of inaccuracy, the dominant one was recorded. Four YouTube Shorts videos rated Inaccurate received no subtype and are shown as not classified. Abbreviation: GLP-1 RA, glucagon-like peptide-1 receptor agonist.

### 3.3 Reach of inaccurate content

The 194 videos assessed for content had accumulated 174.6 million views. Videos rated Accurate accounted for 24.0% of them, Insufficient for 29.5%, and Inaccurate for 38.6%, with hazard-positive videos at 19.2% (**Figure 2C**; **Table 5**). Within each platform, median view counts did not differ across accuracy categories (Kruskal-Wallis p = 0.51 on TikTok and p = 0.23 on YouTube Shorts), so inaccurate videos were not individually more viewed. The imbalance in exposure reflects how few accurate videos there were. It was starkest on YouTube Shorts, where videos rated Accurate made up 39% of the sample but only 16.7% of views.

**Figure 2:**
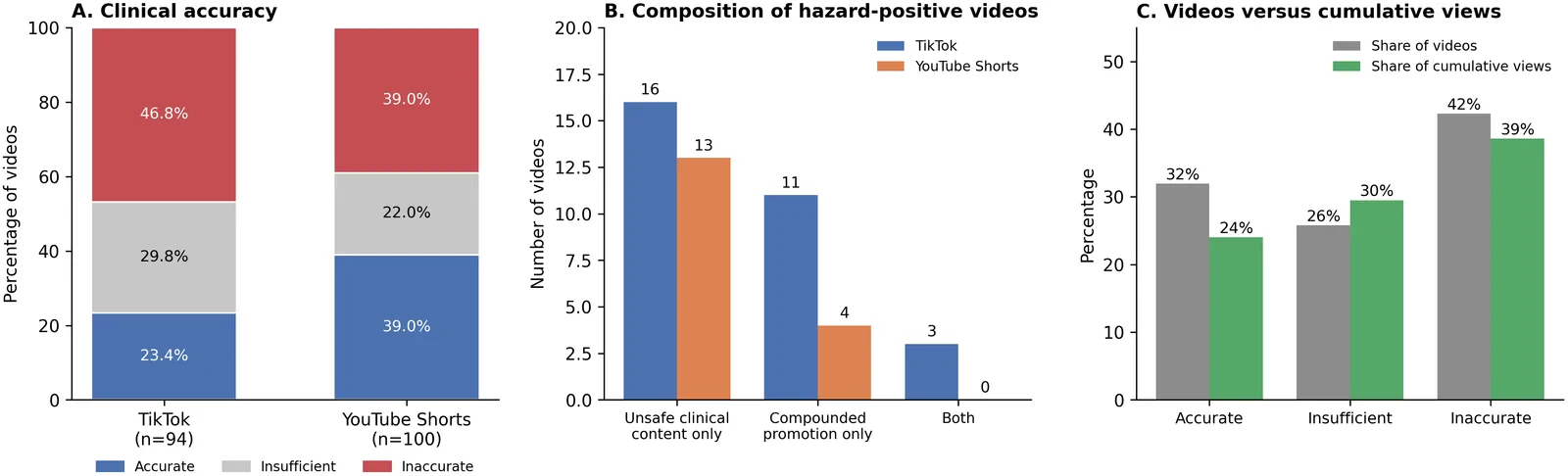
Content quality and reach. **(A)** Distribution of clinical accuracy ratings by platform. **(B)** Composition of hazard-positive videos, separating directly unsafe clinical content from promotion of compounded products. **(C)** Share of videos against share of cumulative views by accuracy rating, both platforms combined.

**Table 5:** Cumulative views by clinical accuracy rating.

Measure	TikTok	YouTube Shorts	Both platforms
Total views of assessed videos	151 583 600	23 029 877	174 613 477
Accurate, % of views	25.1	16.7	24.0
Insufficient, % of views	27.4	43.7	29.5
Inaccurate, % of views	38.5	39.6	38.6
Hazard-positive, % of views	20.3	11.7	19.2
Median views, Accurate	775 250	14 430	–
Median views, Insufficient	943 500	41 783	–
Median views, Inaccurate	1 100 000	33 992	–
Kruskal-Wallis p across accuracy categories	0.51	0.23	–

Percentages are of total views gathered by the assessed videos on each platform. Median view counts did not differ significantly across accuracy categories within either platform.

Restricting both platforms to videos of 60 s or less, the format they share, did not soften the accuracy findings. Among these, 8 of 57 rated TikTok videos were Accurate against 39 of 100 YouTube Shorts (p = 0.002), and hazard potential remained more common on TikTok without reaching significance (26.2% vs 17.0%, p = 0.159). Longer TikTok videos were thus more likely, not less, to carry accurate clinical information (**Supplementary Table 2**).

## 4 Discussion

Short-form videos on incretin-based obesity therapies reach very large audiences, and most of what they carry is either wrong or clinically empty. TikTok and YouTube Shorts contributed equally to the analytic sample, but TikTok videos reached far more people. That advantage is one of distribution rather than of audience response. TikTok videos were reaching much larger audiences in roughly a third of the time, yet viewers who watched a YouTube Short commented on it more often per view. Raw like and comment totals overstate the gap between the platforms, which is why we report normalized rates alongside them. The reach advantage matches findings on other health topics, where TikTok generally produces broader visibility than YouTube Shorts and similar platforms (19). View counts in our sample ran above those reported for other health topics, which says something about the level of public interest in these medications (14, 16).

Across both platforms, most content was either inaccurate or carried no clinical information (TikTok 76.6% [95% CI 66.8-84.7%] and YouTube 61.0% [95% CI 50.7-70.6%]). This matters because the search terms were the names of the medications. Someone looking for information about a drug they have been prescribed is unlikely to find anything clinically reliable. The two categories are not equivalent, though, and should not be pooled under the label of misinformation. Videos rated Inaccurate made incorrect clinical claims. Videos rated Insufficient, most of them weight-loss testimonials with little or no medical explanation, were not necessarily wrong. They were empty. The distinction matters for what to do about it, since correcting a false claim and filling a vacuum call for different responses. Either way, this content can reinforce the belief that weight loss on GLP-1 RA therapy happens effortlessly and without any change in diet or activity.

The exposure analysis sharpens the point. Inaccurate videos were not individually more popular, yet accurate videos captured only about a quarter of the 174.6 million views in our sample, simply because there were so few of them. On YouTube Shorts the gap was widest, at 39% of videos and under 17% of views. Counting videos alone understates how lopsided the information environment is for anyone searching these drug names.

TikTok videos trended toward being less accurate than those on YouTube Shorts, though the difference did not reach significance (p = 0.062). This is consistent with earlier work on health-related short-form video reporting higher quality and accuracy on YouTube (14), and with instrument-based evaluations in other clinical areas (30). The platforms differ in how they police this content. TikTok prohibits health misinformation that could cause harm or delay care, while YouTube ties its medical misinformation policy to guidance from health authorities and operates a channel strike system under which repeated violations can end an account (31, 32). Creator composition may matter as much as policy. YouTube content is more often made by healthcare professionals, whereas TikTok content comes more often from non-expert or lifestyle creators (19). We did not code creator type, and whether it explains the accuracy difference remains open, although account-level metadata for TikTok creators are given in **Supplementary Table 4**.

Videos promoting compounded products deserve separate attention, because they account for the entire platform difference in hazard potential. Set them aside and the two platforms carried similar amounts of directly unsafe clinical advice. What set TikTok apart was that compounded product promotion was four times more common. The risk here is not mainly one of incorrect medical information. It is that viewers are routed toward preparations that fall outside FDA review for potency, purity, and sterility. Compounded use should be reserved for patients who need GLP-1 RA therapy but cannot obtain an FDA-approved formulation, and even then it needs careful medical supervision (20). These formulations also carry risks of improper storage, inaccurate dosing, and confusion between legitimate and fraudulent products (20, 21). One video in our sample promoted a fraudulent preparation sold as a “natural stimulant GLP-1.” The distinction has a practical edge. Content moderation aimed at correcting clinical claims will not touch a marketing channel, whereas enforcement of pharmaceutical advertising and unapproved-product rules would.

Some videos gave explicit dosing errors, including one recommending 40 units of tirzepatide, or wrong administration instructions, such as injecting into the thigh for “fewer side effects” with no evidence behind it. Others gave non-evidence-based self-management advice, such as using “Liquid IV” for fatigue. We reported this content to the platforms and recommended removal.

Stigmatizing content appeared in a subset of videos, 13 of 94 on TikTok (13.8%) and 7 of 100 on YouTube Shorts (7.0%). Examples included derogatory or dismissive language such as “fat,” “skinny,” “thin,” “fluffy,” and “hangry woman,” along with phrases implying that people with obesity should simply “eat less.” Such portrayals reinforce negative stereotypes and can discourage people with obesity from seeking care (22–24). Weight stigma carries measurable psychological and behavioral consequences, and its persistence in this content is worth watching.

Reviewers identified commercial influence in 17 TikTok and 14 YouTube Shorts videos, yet none of the videos in our sample carried a platform sponsorship disclosure tag in the captured metadata. Both platforms require creators to declare paid partnerships. The gap between promotion a physician can recognize and promotion that is formally declared suggests these rules are not reaching this content.

Social media also does something useful here, and the risks should not obscure it. These platforms give patients a place to find each other and ask questions away from clinical settings that can feel distant or judgmental. That matters for people living with obesity, who often meet implicit bias in care. It also points to an opening. Trusted patient voices and influencers could carry evidence-based information in formats people actually watch. The Center for Health Communication at the Harvard T.H. Chan School of Public Health has done this in mental health, working with TikTok creators to reach young audiences (25). The same approach in obesity could spread accurate information without stripping out the authenticity that draws patients to these platforms in the first place.

This study has several limitations. No validated instrument exists for judging the quality of short-form health video (26, 27). DISCERN, the Global Quality Score, and the JAMA benchmark criteria have been used on longer YouTube videos but do not transfer to this format, so we did not use them (26–30). Videos were coded once by a single physician reviewer with adjudication by a senior reviewer rather than in duplicate, so inter-rater agreement could not be calculated and the subjective element in content evaluation cannot be quantified. The free-text subtype labels also suggest reviewers applied the guide somewhat differently across platforms, and four YouTube Shorts videos rated Inaccurate were left without a subtype. Sampling was built on six medication-name keywords rather than on the platforms as a whole, so the sample reflects what those searches surfaced. The two platforms impose different length limits, and TikTok videos ran up to 432 s while YouTube Shorts were capped at 60 s, though restricting the comparison to videos of 60 s or less did not change our conclusions. Videos were nested within creators, with up to 3 per creator on TikTok and 7 on YouTube Shorts, and our analyses did not account for that clustering. Only English-language videos were included, and non-English content would be needed to see how this misinformation travels across languages. We did not extract comment text, creator credentials, closed-captioning status, or whether videos cited sources, and the metadata available to us did not include these fields for YouTube Shorts. Finally, this is a snapshot from a single day, and what circulates on these platforms changes quickly.

## 5 Conclusions

We examined short-form video content on incretin-based obesity therapies across TikTok and YouTube Shorts. TikTok reached substantially larger audiences, but on both platforms most content was clinically inaccurate or carried no meaningful clinical information, and accurate videos drew only about a quarter of the cumulative views in our sample. Promotion of compounded products was concentrated on TikTok and accounted for the platform difference in hazard potential. Stigmatizing portrayals of obesity appeared on both.

Social media now shapes how the public understands obesity treatment, which makes accurate, stigma-free, and transparent communication about these medications a practical concern rather than an abstract one. Clinicians, professional societies, and the platforms themselves will each have a part in curbing misinformation and limiting commercial influence. Our findings point to two separate targets. One is the near-absence of accurate clinical content in what viewers actually see. The other is the use of these platforms as a marketing channel for products that fall outside regulatory review.

## Data Availability

The raw data supporting the conclusions of this article will be made available by the authors, without undue reservation.
