# Supplementary material for: Accuracy, potential harm, weight stigma, and commercial influence in short-form social media content on incretin-based therapies for obesity
Source: Front Endocrinol (Lausanne). 2026 Oct 2;17:1844311. doi: 10.3389/fendo.2026.1844311 (PMC13671960; doi:10.3389/fendo.2026.1844311)
Supplement: Supplementary file 1 [file Table1.docx]

**Supplementary Material**

Accuracy, Potential Harm, Weight Stigma, and Commercial Influence in Short-Form Social Media Content on Incretin-Based Therapies for Obesity

Manuscript ID: 1844311

**Supplementary Table 1. Included videos per search keyword**

| **Keyword** | **TikTok (n = 100)** | **YouTube Shorts (n = 100)** |
| --- | --- | --- |
| Ozempic | 25 | 22 |
| Semaglutide | 20 | 15 |
| Tirzepatide | 18 | 21 |
| Mounjaro | 16 | 15 |
| Wegovy | 14 | 11 |
| Zepbound | 7 | 16 |
| Total | 100 | 100 |

*Each video is attributed to the keyword under which it was first retrieved. Videos returned by more than one keyword were counted once. The uneven distribution reflects differences in how many high-view videos each keyword surfaced rather than a sampling quota.*

**Supplementary Table 2. Sensitivity analysis restricted to videos of 60 seconds or shorter**

|  | **TikTok (n = 61)** | **YouTube Shorts (n = 100)** | **p value** |
| --- | --- | --- | --- |
| Clinical accuracy |  |  | 0.002 |
| Accurate | 8 (14.0) | 39 (39.0) |  |
| Insufficient | 23 (40.4) | 22 (22.0) |  |
| Inaccurate | 26 (45.6) | 39 (39.0) |  |
| Hazard potential (composite) | 16 (26.2) | 17 (17.0) | 0.159 |

*Values are number (%). Accuracy percentages are of the 57 videos in this subgroup with a content rating. All YouTube Shorts videos in the sample were 60 s or shorter, whereas 39 TikTok videos exceeded this limit. Restricting the comparison to the format common to both platforms did not attenuate the accuracy findings, indicating that the longer permissible length on TikTok does not explain the platform difference.*

**Supplementary Table 3. Country of origin of TikTok videos**

| **Country** | **Videos (n = 100)** |
| --- | --- |
| United States | 83 |
| United Kingdom | 10 |
| Canada | 3 |
| Australia | 1 |
| Brazil | 1 |
| Greece | 1 |
| Ghana | 1 |

*Country of creation as recorded in TikTok video metadata. The equivalent field was not available for YouTube Shorts. Most content therefore originated in a jurisdiction that permits direct-to-consumer prescription drug advertising, while circulating in jurisdictions that do not.*

**Supplementary Table 4. Creator characteristics, TikTok**

|  | **TikTok (n = 100)** |
| --- | --- |
| Distinct creators | 92 |
| Maximum videos per creator | 3 |
| Verified accounts | 12 |
| Follower count, median (Q1–Q3) | 42 550 (8 843–293 800) |

*Creator type was not coded prospectively. These objective account-level metadata are reported in response to reviewer comments on creator composition. On YouTube Shorts, 100 videos came from 73 creators, with a maximum of 7 videos from a single creator; equivalent account metadata were not captured for that platform.*

**Supplementary Methods. Coding definitions**

Clinical accuracy. Rated as Accurate when the medical information presented was consistent with current evidence and product labeling; Inaccurate when the video contained clinical claims that were incorrect or unsupported; and Insufficient when the video contained no meaningful clinical information, most often a weight-loss testimonial without medical explanation. Insufficient denotes absence of clinical content rather than the presence of incorrect content.

Hazard potential. Information that, if acted upon, could plausibly prompt a behavioral change leading to direct medical harm. Two pathways led to a hazard-positive classification. The first was the reviewer’s direct judgment that the content was unsafe, for example an explicit dosing error. The second was an a priori rule classifying promotion of a compounded product as hazard-positive by definition. Results are reported under both the composite definition and the direct-judgment definition alone.

Compounded medications. GLP-1 RA or GIP/GLP-1 RA preparations produced by compounding pharmacies or other non-manufacturer sources rather than dispensed as an FDA-approved branded product, including peptide preparations sold for research use only and products marketed as natural or herbal GLP-1 alternatives.

Obesity stigma. Weight-related bias, negative body image, or shaming expressions, including derogatory or dismissive language about body size and framing that attributes obesity to personal failure.

Commercial influence. Explicit or implicit promotion of a product, clinic, influencer brand, or service, including sponsorships, discount codes, or external links.

Thematic subtypes of inaccurate content. Applied as mutually exclusive categories. Where a video contained more than one type of inaccuracy, the reviewer recorded the dominant one.

**Supplementary Data**

The video-level dataset supporting this analysis, containing platform, video identifier, metadata, and the four domain ratings for all 200 included videos, is available from the corresponding author on reasonable request.
